# Supplementary material for: Pancreatic Ductal Cell-Derived Extracellular Vesicles Are Effective Drug Carriers to Enhance Paclitaxel’s Efficacy in Pancreatic Cancer Cells through Clathrin-Mediated Endocytosis
Source: Int J Mol Sci. 2022 Apr 26;23(9):4773. doi: 10.3390/ijms23094773 (PMC9099870; doi:10.3390/ijms23094773)
Supplement: Supplementary file 1 [file ijms-23-04773-s001.zip › ijms-1693944-supplementary.pdf]

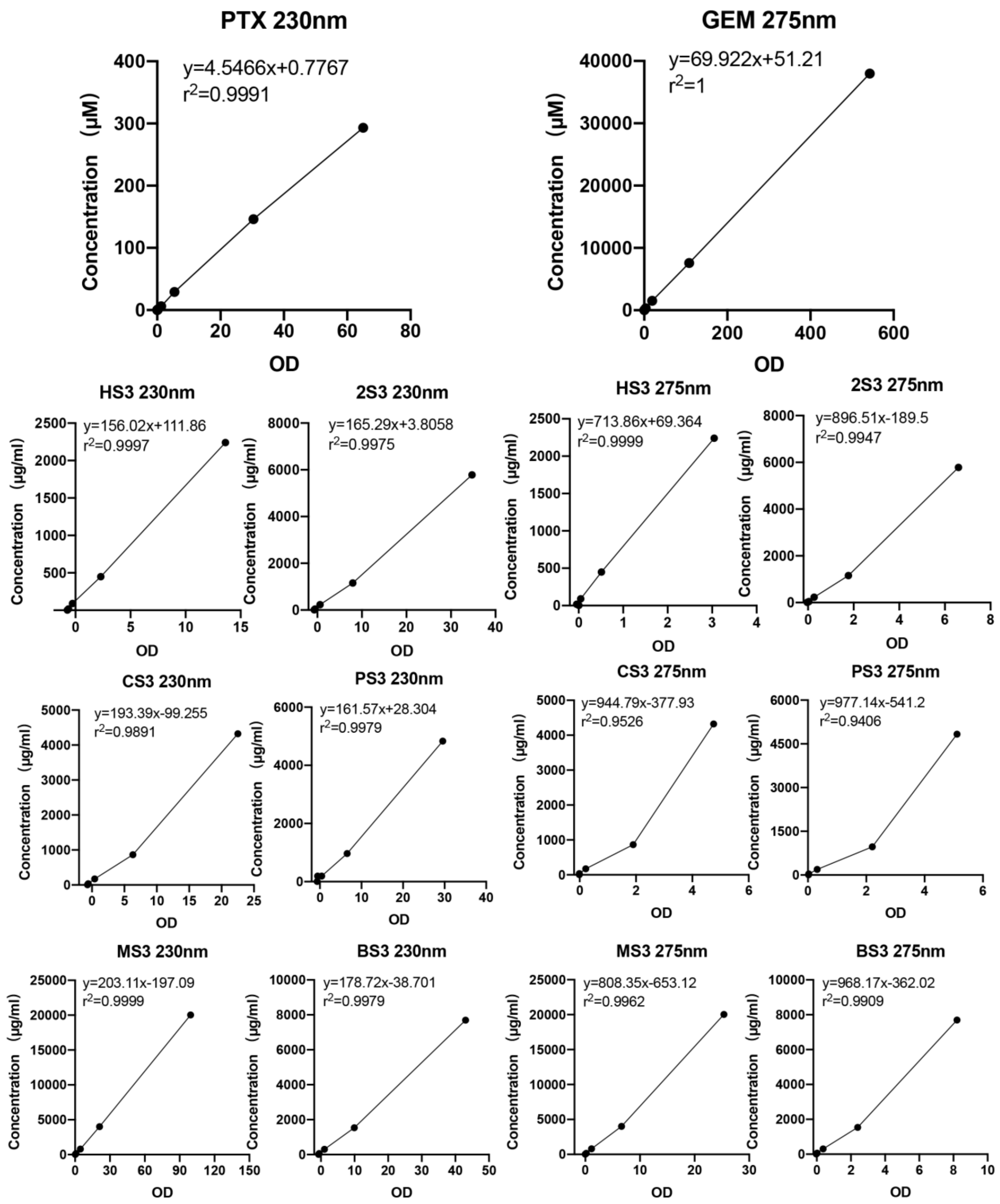

Figure S1. Standard curves of absorbencies at 230 nm or 275 nm for free PTX, GEM and sEVs derived from 6 human cell lines**.**

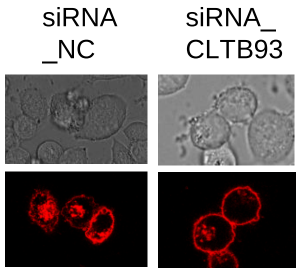

Figure S2. Knockdown of clathrin light chain impairs transferrin internalization. PANC-1 cells were transfected with siRNA_NC and siRNA_CLTB93 (see Table 1). Twenty four hours post transfection, the TfR-pHuji plasmid (Addgene Plasmid #61505) was introduced to the cells, and confocal images were taken 72 hours post siRNA transfection (Excitation, 488 nm, emission, 588 nm, Leica SP8 Confocal White Light Laser system microscope using a 40X objective). Knockdown of clathrin light chain reduced intracellular transferrin levels.
